# Development and Validation of a Nomogram to Predict the Probability of Breast Cancer Pathologic Complete Response after Neoadjuvant Chemotherapy: A Retrospective Cohort Study

**DOI:** 10.3389/fsurg.2022.878255

**Published:** 2022-06-09

**Authors:** Yijun Li, Jian Zhang, Bin Wang, Huimin Zhang, Jianjun He, Ke Wang

**Affiliations:** Department of Breast Surgery, the First Affiliated Hospital of Xi’an Jiaotong University, Xi’an, China

**Keywords:** breast cancer, neoadjuvant chemotherapy, nomogram, clinical cancer research, pathologic complete response

## Abstract

**Background:**

The methods used to predict the pathologic complete response (pCR) after neoadjuvant chemotherapy (NAC) have some limitations. In this study, we aimed to develop a nomogram to predict breast cancer pCR after NAC based on convenient and economical multi-system hematological indicators and clinical characteristics.

**Materials and Methods:**

Patients diagnosed from July 2017 to July 2019 served as the training group (*N* = 114), and patients diagnosed in from July 2019 to July 2021 served as the validation group (*N* = 102). A nomogram was developed according to eight indices, including body mass index, platelet distribution width, monocyte count, albumin, cystatin C, phosphorus, hemoglobin, and D-dimer, which were determined by multivariate logistic regression. Internal and external validation curves are used to calibrate the nomogram.

**Results:**

The area under the receiver operating characteristic curve was 0.942 (95% confidence interval 0.892–0.992), and the concordance index indicated that the nomogram had good discrimination. The Hosmer–Lemeshow test and calibration curve showed that the model was well-calibrated.

**Conclusion:**

The nomogram developed in this study can help clinicians accurately predict the possibility of patients achieving the pCR after NAC. This information can be used to decide the most effective treatment strategies for patients.

## Introduction

Breast cancer is the most common malignant tumor observed in females worldwide (1). Neoadjuvant chemotherapy (NAC) is widely used and has important implications for patients with locally advanced breast cancer. In these patients, NAC can reduce tumor diameter and staging, which increases the possibility of operation or breast-conserving surgery and reduces the necessity for axillary lymph node dissection. Although it is still controversial whether NAC can provide long-term survival benefits to patients (2–4), the pathological complete response (pCR) can be used as an alternative index of disease-free survival and overall survival (5–7).

The prediction of the pCR before the start of NAC is very important for clinicians to formulate individualized and precise treatment plans for patients. Firstly, for patients with a low possibility of reaching pCR, abandoning NAC and choosing surgery as soon as possible can not only avoid the toxic effects related to chemotherapy, and save medical costs and resources, but also avoid the time delay caused by NAC, resulting in disease progression. Furthermore, the study of predictors of NAC efficacy will bring new directions for the molecular mechanism of tumorigenesis and chemosensitivity.

Nomograms are widely used to quantify risk in various diseases using intuitive charts combined with known prognostic factors (8). At present, there are some nomograms models for the prediction of pCR in China’s breast cancer population. The methods used to predict pCR include clinicopathological features (9, 10), molecular biomarker analysis (11), medical imaging (12) et al. However, the prediction accuracy of traditional clinicopathological indexes is not satisfactory, while genetic and imaging indexes are expensive, complicated, and have limited application in clinical practice. In recent years, scientists have tried to use common hematological indicators to predict the pCR (13, 14). For example, carbohydrate antigen 153 was proven to be a reliable independent predictor of the pCR (15). Hematological indicators are convenient and economical, but the prediction efficiency of a single indicator is low, and few studies have comprehensively evaluated the combined predictive value of various hematological indicators for the pCR.

In this study, we collected multi-system hematological and clinical characteristics before NAC, and analyzed the relationship between these indicators and NAC efficacy. Finally, we developed a nomogram to predict the possibility of achieving pCR in breast cancer patients.

## Materials and Methods

### Patients and Ddata Collection

Patients with breast cancer who underwent NAC and surgical treatment at the First Affiliated Hospital of Xi’an Jiaotong University from July 2017 to July 2021 were retrospectively enrolled. Neoadjuvant chemotherapy drugs are based on taxol and / or anthracyclines. Targeted therapeutic drugs were added for all HER2-positive patients. Formulation of the treatment schedule for all patients was determined in accordance with the 2019 version of the National Comprehensive Cancer Network (NCCN) Breast Cancer Clinical Practice Guidelines (16) and discussed by all clinicians in the department.

The inclusion criteria were as follows: (1) invasive breast cancer diagnosed by biopsy; (2) 18–70 years of age; (3) clinical stage II or III breast cancer; (3) fulfillment of the requirements of 2019 NCCN guidelines for NAC for breast cancer (16); (4) completion of all courses of standardized NAC and surgical treatment according to the established scheme. Patients with the following characteristics were excluded: (1) bilateral breast cancer or a second primary cancer; (2) distant metastases identified during treatment; (3) incomplete pathological or hematological examination results; and (4) refusal to participate in the relevant clinical studies. (5) patients with acute diseases (such as acute infection, acute organ dysfunction, etc.) (6) Patients with chronic diseases (such as chronic renal dysfunction, chronic liver dysfunction, chronic coagulopathy, chronic blood system dysfunction, etc.) but not controlled and stable. (7) patients who terminated chemotherapy or changed chemotherapy regimen or cycle due to chronic diseases.

We collected information on patients’ clinical characteristics and hematological indicators from the electronic medical records system. Clinical characteristics included age, clinical stage, clinical tumor stage, clinical lymph node stage, chemotherapy regimen, chemotherapy cycle, menstrual status, molecular typing, estrogen receptor (ER) state, progesterone receptor (PR) state, Ki-67 index, human epidermal growth factor receptor 2 (HER-2) state, and body mass index (BMI). Hematological tests were carried out to assess liver function, renal function, electrolyte concentrations, complete blood counts, and coagulation function. The total number of indicators measured was 61. BMI was calculated by dividing body weight in kilograms by the square of height (m^2^). Testing for all clinical characteristics and hematological indicators was completed within three days before patients underwent NAC.

The clinical stage was determined by the American Joint Committee on Cancer (17) clinical stage guidelines (8th edition). ER and PR expression of <1% was considered negative by immunohistochemical staining. HER-2 status was determined in accordance with the American Society of Clinical Oncology/College of American Pathologists guidelines (18). Molecular typing was determined by St. Gallen guidelines (19). According to NCCN guidelines [20], pCR was defined as histological evidence that no invasive tumor was found in primary breast lesions or axillary lymph nodes after NAC, regardless of the presence of residual ductal carcinoma in situ (ypT0/isypN0).

It is a retrospective study and the study was approved by the Ethics Committee of the First Affiliated Hospital of Xi’an Jiaotong University.

A complete blood count was conducted using the BC-5390 hematology analyzer (Mindray, Shenzhen, China); the coagulation function test was conducted using the CA7000 automatic hemagglutination apparatus (Sysmex, Kobe, Japan); and liver function, renal function, and electrolyte tests were conducted using the VITROS 5600 automatic biochemical immune analyzer (JNJ, New Jersey, USA).

### Statistical Analysis

Patients diagnosed from 2017 to 2019 served as the training group, and patients diagnosed from 2019 to 2021 served as the validation group. Only age was analyzed as a continuous variable, while all other clinical characteristics and hematological indicators were analyzed as categorical variables. Continuous variables other than age were divided into two groups according to the optimal cut-off value, which was obtained by calculating the maximum Youden’s index (sensitivity + specificity − 1) using the receiver operating characteristic (ROC) curve (20). The Chi-squared test or the Mann–Whitney U test was performed to analyze the correlation between pCR and clinical characteristics and hematological indicators. If the expected frequency was <5, Fisher’s exact test was used for analysis. All variables demonstrating a *p*-value of <0.1 in the univariate analysis were entered into the forward stepwise logistic regression (likelihood ratio) to identify independent predictors, and forest plots were drawn based on the results of multivariate logistic regression. According to the results of logistic regression, a nomogram was established. Internal and external validation curves are used to calibrate the nomogram. A Hosmer–Lemeshow test was performed to check the goodness of fit of the model. The ROC curve, area under the curve (AUC), and concordance index (C-index) were used to display the discrimination of the nomogram. IBM SPSS Statistics (version 22.0; IBM Corporation, Armonk, NY, USA) and R software (version 3.6.2; The R Foundation for Statistical Computing, Austria, Vienna) were used to perform statistical analysis. All p values were two-sided and a *p*-value of <0.05 was considered statistically significant.

## Results

### Clinical Characteristics of Patients and the pCR After NAC

A total of 114 eligible patients were enrolled in the training group, and 102 patients were in the validation group. The clinical characteristics of training group were shown in Table 1. The average age of training group patients was 47.9 years. Stage II and stage III breast cancer accounted for 76.3% (87/114) and 23.7% (27/114) of all breast cancers, respectively. Of all patients, 72.8% (83/114) were cT2, 69.3% (79/114) were cN1, 85.1% (97/114) underwent ≥6 chemotherapy cycles, and 56.1% (64/114) were premenopausal. As for the molecular subtype, 43.9% (50/114) of cases were HER-2 positive molecular subtype, 50.9% (58/114) were ER negative, 74.6% (85/114) were PR negative, and 76.3% (87/114) had HER2 expression positivity. The optimal cut-off value was 27.5% for the Ki-67 index and 21.281 kg/m^2^ for BMI. Among the clinical characteristics, a lower clinical stage and a lower BMI correlated with a higher pCR rate (*p* < 0.1). There was no statistical significance between age, cT stage, cN stage, chemotherapy cycle, chemotherapy regimen, menstrual status, molecular subtype, ER state, PR state, Ki-67 index, HER-2 state, and pCR rate. The clinical characteristics of patients in validation group were shown in Supplementary Table S1.

**Table 1:** Clinical characteristics of 114 patients with breast cancer and their correlation with the pathologic complete response rate after neoadjuvant chemotherapy.

Factors	Total (%)	pCR (%)	Non-pCR (%)	*p* value
Age (years, mean ± SD)	47.9 ± 10.3	47.3 ± 10.1	48.2 ± 10.5	0.558
Clinical stage				**0.098**
II	87 (76.3)	27 (31.0)	60 (69.0)	
III	27 (23.7)	4 (14.8)	23 (85.2)	
Clinical tumor stage				0.685
cT1	15 (13.2)	4 (26.7)	11 (73.3)	
cT2	83 (72.8)	22 (26.5)	61 (73.5)	
cT3	13 (11.4)	5 (38.5)	8 (61.5)	
cT4	3 (2.6)	0 (0.0)	3 (100.0)	
Clinical nodal stage				0.498
cN0	11 (9.6)	4 (36.4)	7 (63.6)	
cN1	79 (69.3)	23 (29.1)	56 (70.9)	
cN2	19 (16.7)	4 (21.1)	15 (78.9)	
cN3	5 (4.4)	0 (0.0)	5 (100.0)	
Chemotherapy regimen				0.717
TAC/ AC-T/ TA	62 (47.7)	26 (41.9)	36 (58.1)	
TC/TX/TP/AC	10 (7.7)	7 (70.0)	3 (30.0)	
AC-TH/ TCbH	38 (29.2)	17 (44.7)	21 (55.3)	
TCbHP/ THP/ AC-THP	20 (15.4)	8 (40.0)	12 (60.0)	
Chemotherapy cycle				
<6	17 (14.9)	3 (17.6)	14 (82.4)	0.555
≥6	97 (85.1)	28 (28.9)	69 (71.1)	
Menopausal status				0.271
Premenopausal	64 (56.1)	20 (31.3)	44 (68.8)	
Peri/postmenopausal	50 (43.9)	11 (22.0)	39 (78.0)	
Molecular subtype				0.659
Luminal B (Her2−)	7 (6.1)	2 (28.6)	5 (71.4)	
Luminal B (Her2+)	37 (32.5)	11 (29.7)	26 (70.3)	
Her2 positive	50 (43.9)	11 (22.0)	39 (78.0)	
Triple negative	20 (17.5)	7 (35.0)	13 (65.0)	
Estrogen receptor				0.174
Negative	58 (50.9)	19 (32.8)	39 (67.2)	
Positive	56 (49.1)	12 (21.4)	44 (78.6)	
Progesterone receptor				0.362
Negative	85 (74.6)	25 (29.4)	60 (70.6)	
Positive	29 (25.4)	6 (20.7)	23 (79.3)	
HER-2				0.412
Negative	27 (23.7)	9 (33.3)	18 (66.7)	
Positive	87 (76.3)	22 (25.3)	65 (74.7)	
Ki-67,%				0.108
<27.5	14 (12.3)	1 (7.1)	13 (92.9)	
≥27.5	100 (87.7)	30 (30.0)	70 (70.0)	
Body mass index, kg/m^2^				**0.073**
<21.281	24 (21.1)	10 (41.7)	14 (58.3)	
≥21.281	90 (78.9)	21 (23.3)	69 (76.7)	

*NAC, Neoadjuvant chemotherapy; pCR, Pathologic complete response; HER2, Human epidermal growth factor receptor 2.*

*Bold values mean p < 0.1.*

### Correlation Between Hematological Indicators and pCR

The correlation between pCR and 61 hematological indicators was analyzed (Supplementary Table S2). Twenty-three indicators correlated with the pCR rate (*p* < 0.1). The pCR was more likely to be achieved in patients who had high values for alkaline phosphatase (ALP), albumin (ALB), total protein (TP), creatinine (CRE), cystatin C (Cys-C), calcium (Ca^2+^), hemoglobin (HGB), red blood cell (RBC) count, mean platelet volume (MPV), fibrinogen content (FIB), platelet large cell ratio (P-LCR), activated partial thromboplastin time (APTT), and D-dimer (D-D). Conversely, potassium (K^+^), phosphorus (POH), mean corpuscular hemoglobin concentration (MCHC), platelet distribution width (PDW), monocyte count (MONO), monocyte percentage (MONO%), thrombin time (TT), and thrombin time ratio (TT R) were negatively correlated with pCR (Table 2).

**Table 2:** Hematological indicators related to the pathologic complete response rate after neoadjuvant chemotherapy in 114 patients with breast cancer.

Factors	Total (%)	pCR (%)	Non-pCR (%)	*P* value
Liver function test				
Alkaline phosphatase, U/L				**0.083**
<61.5	32 (28.1)	5 (15.6)	27 (84.4)	** **
≥61.5	82 (79.9)	26 (31.7)	56 (68.3)	** **
Total protein, g/L				**0.073**
<79.45	90 (78.9)	21 (23.3)	69 (76.7)	** **
≥79.45	24 (21.1)	10 (41.7)	14 (58.3)	** **
Albumin, g/L				**0.031**
<47.45	83 (72.8)	18 (21.7)	65 (78.3)	** **
≥47.45	31 (27.2)	13 (41.9)	18 (58.1)	** **
Creatinine, μmol/L				**0.069**
<43.5	24 (21.2)	3 (12.5)	21 (87.5)	** **
≥43.5	90 (78.9)	28 (31.1)	62 (68.9)	** **
Cystatin C, mg/L				**<0.001**
<1.05	104 (91.2)	23 (22.1)	81 (77.9)	** **
≥1.05	10 (8.8)	8 (80.0)	2 (20.0)	** **
Electrolyte test				
Potassium, mmol/L				**0.704**
<4.34	95 (83.3)	29 (30.5)	66 (69.5)	** **
≥4.34	19 (16.7)	2 (10.5)	17 (89.5)	** **
Phosphorus, mmol/L				**0.010**
<1.135	66 (57.9)	24 (36.4)	42 (63.6)	** **
≥1.135	48 (42.1)	7 (14.6)	41 (85.4)	** **
Calcium, mmol/L				**0.030**
<2.325	63 (55.3)	12 (19.0)	51 (81.0)	** **
≥2.325	51 (44.7)	19 (37.3)	32 (62.7)	** **
Complete blood count				
Red blood cell count/L				**0.046**
<5.04	108 (94.7)	27 (25.0)	81 (75.0)	** **
≥5.04	6 (5.3)	4 (66.7)	2 (33.3)	** **
Hemoglobin, g/L				**0.067**
<147.5	103 (90.4)	25 (24.3)	78 (75.7)	** **
≥147.5	11 (9.6)	6 (54.5)	5 (45.5)	** **
Mean corpuscular hemoglobin concentration, g/L		0.065		** **
<323.5	99 (86.8)	30 (30.3)	69 (69.7)	** **
≥323.5	15 (13.2)	1 (6.7)	14 (93.3)	** **
Platelet distribution width, fL				**0.028**
<14.85	58 (50.9)	21 (36.2)	37 (63.8)	** **
≥14.85	56 (49.1)	10 (17.9)	46 (82.1)	** **
Mean platelet volume, fL				**0.051**
<10.7	34 (29.8)	5 (14.7)	29 (85.3)	** **
≥10.7	80 (70.2)	26 (32.5)	54 (67.5)	** **
Platelet large cell ratio, %				**0.068**
<31.5	37 (32.5)	6 (16.2)	31 (83.8)	** **
≥31.5	77 (67.5)	25 (32.5)	52 (67.5)	** **
Monocyte count, 10^9^/L				**0.004**
<0.185	15 (13.2)	9 (60.0)	6 (40.0)	** **
≥0.185	99 (86.8)	22 (22.2)	77 (77.8)	** **
Monocyte percentage, %				**0.020**
<3.55	21 (18.4)	10 (47.6)	11 (52.4)	** **
≥3.55	93 (81.6)	21 (22.6)	72 (77.4)	** **
Coagulation function test				
Activated partial thromboplastin time, s				**0.088**
<35.95	59 (51.8)	12 (20.3)	47 (79.7)	** **
≥35.95	55 (48.2)	19 (34.5)	36 (65.6)	** **
Thrombin time, s				**0.013**
<16.75	75 (65.8)	26 (34.7)	49 (65.3)	** **
≥16.75	39 (34.2)	5 (12.8)	34 (87.2)	** **
Thrombin time ratio				**0.013**
<0.985	75 (65.8)	26 (34.7)	49 (65.3)	** **
≥0.985	39 (34.2)	5 (12.8)	34 (87.2)	** **
Fibrinogen content, g/L				**0.022**
<3.085	64 (56.1)	12 (18.8)	52 (81.3)	** **
≥3.085	50 (43.9)	19 (38.0)	31 (62.0)	** **
D-dimer, mg/L				**<0.001**
<0.55	83 (72.8)	15 (18.1)	68 (81.9)	
≥0.55	31 (27.2)	16 (51.6)	15 (48.4)	

*NAC, Neoadjuvant chemotherapy; pCR, Pathologic complete response.*

*Bold values mean p < 0.1.*

### Logistic Regression Analysis of Clinical Characteristics, Hematological Indicators, and pCR

**Figure 1:**
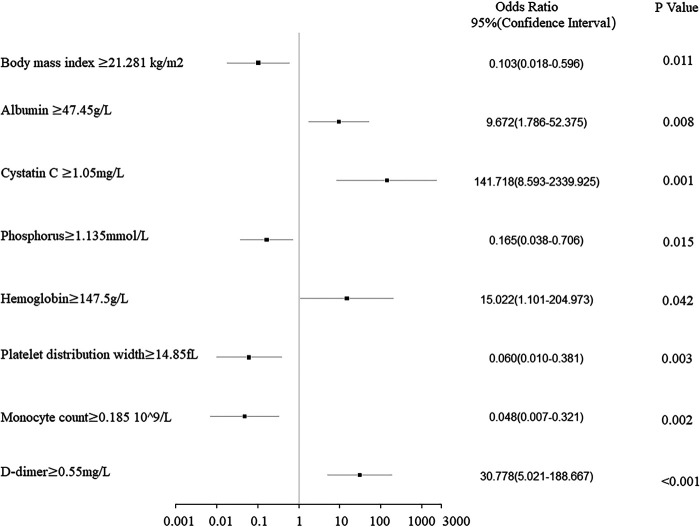
Forest plot analysis of the clinical characteristics and hematological indicators of 114 breast cancer patients related to the pathologic complete response rate after neoadjuvant chemotherapy.

The clinical characteristics and hematological indicators with a *p*-value of <0.1 in the Chi-squared test, Mann–Whitney U test, or Fisher’s exact test were included in the logistic regression analysis. Univariate logistic analyses showed similar results, in which patients with low values for clinical stage, BMI, K^+^, POH, MCHC, PDW, MONO, MONO%, TT, and TT R; and high ALP, TP, ALB, CRE, Cys-C, Ca^2+^, RBC, HGB, MPV, P-LCR, APTT, FIB, and D-D had a higher pCR rate (Table 3). Moreover, multivariate logistic regression showed that BMI, ALB, Cys-C, POH, HGB, PDW, MONO, and D-D were independent predictors for pCR (Table 3). Of these factors, ALB, Cys-C, HGB, and D-D were positively correlated with the pCR rate, while BMI, Cys-C, POH, PDW, and MONO were negatively correlated with the pCR rate (Figure 1).

**Table 3:** Logistic regression analysis of clinical characteristics and hematological indicators related to the pathologic complete response rate after neoadjuvant chemotherapy in 114 patients with breast cancer.

Factors	Univariate analysis			Multivariate analysis		
	OR	95% CI	*p* value	OR	95% CI	*p* value
Clinical stage						
II	1					
III	0.386	0.122–1.226	0.107			
Body mass index, kg/m^2^						
<21.281	1			1		
≥21.281	0.426	0.165–1.099	0.078	0.103	0.018–0.596	0.011
Liver function test						
Alkaline phosphatase, U/L						
<61.5	1					
≥61.5	2.507	0.867–7.248	0.090			
Total protein, g/L						
<79.45	1					
≥79.45	2.347	0.901–6.051	0.078			
Albumin, g/L						
<47.45	1			1		
≥47.45	2.608	1.077–6.313	0.034	9.672	1.786–52.375	0.008
Creatinine, μmol/L						
<43.5	1					
≥43.5	3.161	0.871–11.477	0.080			
Cystatin C, mg/L						
<1.05	1			1		
≥1.05	14.087	2.796–70.984	0.001	141.718	8.593–2339.925	0.001
Electrolyte test						
Potassium, mmol/L						
<4.34	1					
≥4.34	0.268	0.058–1.235	0.091			
Phosphorus, mmol/L						
<1.135	1			1		
≥1.135	0.299	0.116–0.769	0.012	0.165	0.038–0.706	0.015
Calcium, mmol/L						
<2.325	1					
≥2.325	2.523	1.082–5.887	0.032			
Complete blood count						
Red blood cell count/L						
<5.04	1					
≥5.04	6.000	1.040–34.609	0.045			
Hemoglobin, g/L						
<147.5	1			1		
≥147.5	3.744	1.052–13.324	0.042	15.022	1.101–204.973	0.042
Mean corpuscular hemoglobin concentration, g/L						
<323.5	1					
≥323.5	0.164	0.021–1.307	0.088			
Platelet distribution width, fL						
<14.85	1			1		
≥14.85	0.383	0.161–0.913	0.030	0.060	0.010–0.381	0.003
Mean platelet volume, fL						
<10.7	1					
≥10.7	2.793	0.969–8.045	0.057			
Platelet large cell ratio, %						
<31..5	1					
≥31.5	2.484	0.918–6.724	0.073			
Monocyte count, 10^9^/L						
<0.185	1			1		
≥0.185	0.190	0.061–0.593		0.048	0.007–0.321	0.002
Monocyte percentage,%						
<3.55	1					
≥3.55	0.321	0.120–0.859	0.024			
Coagulation function test						
Activated partial thromboplastin time, s						
<35.95	1					
≥35.95	2.067	0.890–4.803	0.091			
Thrombin time, S						
<16.75	1					
≥16.75	0.277	0.097–0.794	0.017			
Thrombin time ratio						
<0.985	1					
≥0.985	0.277	0.097–0.794	0.017			
Fibrinogen content, g/L						
<3.085	1					
≥3.085	2.656	1.137–6.025	0.024			
D-dimer, mg/L						
<0.55	1			1		
≥0.55	4.836	1.967–11.885	0.001	30.778	5.021–188.667	<0.001

*OR, Odds ratio; CI, Confidence interval; NAC, Neoadjuvant chemotherapy; pCR, Pathologic complete response.*

### Development and Validation of the Multi-Factor-Based Nomogram Prediction Model

**Figure 2:**
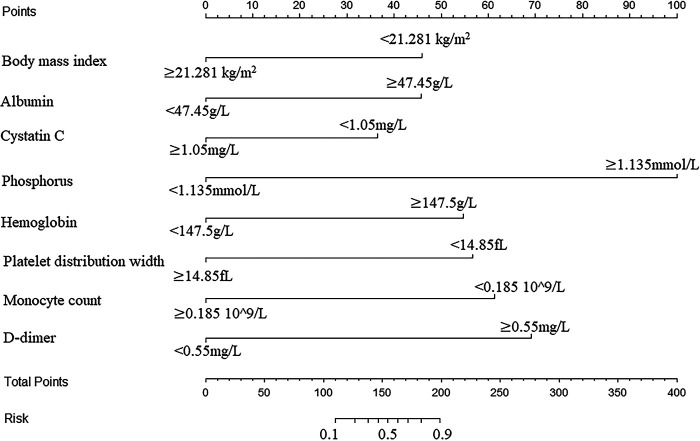
Nomogram to predict the pathologic complete response rate of breast cancer patients after neoadjuvant chemotherapy based on clinical characteristics and hematological indicators.

**Figure 3:**
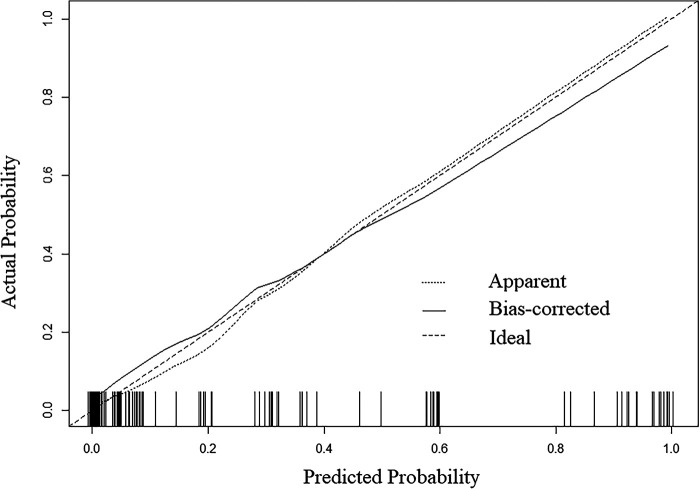
The 1000-bootstrap resampling internal verification curve for the nomogram of pathologic complete response after neoadjuvant chemotherapy in breast cancer patients.

**Figure 4:**
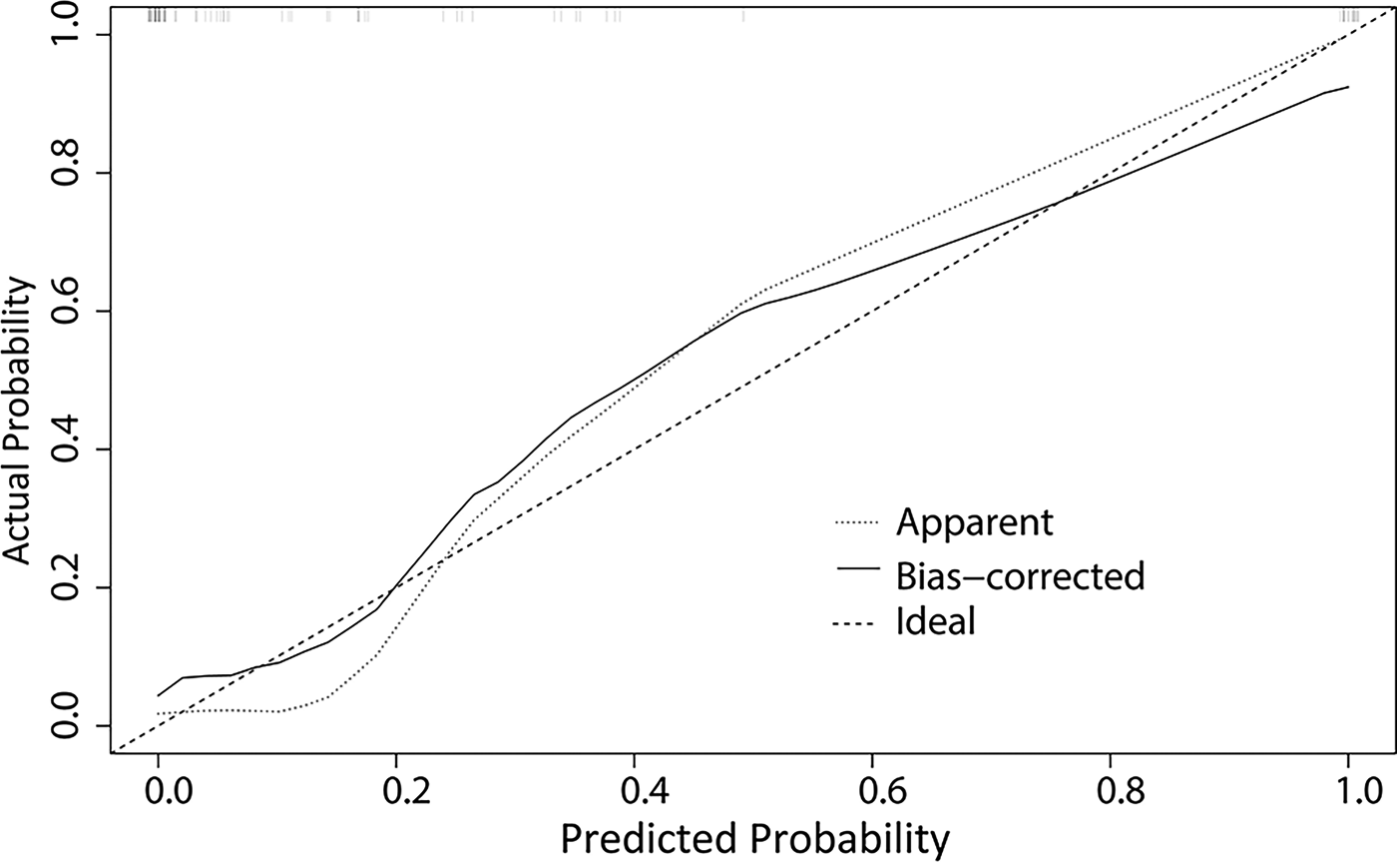
The external validation curve for the nomogram of pathologic complete response after neoadjuvant chemotherapy in breast cancer patients.

**Figure 5:**
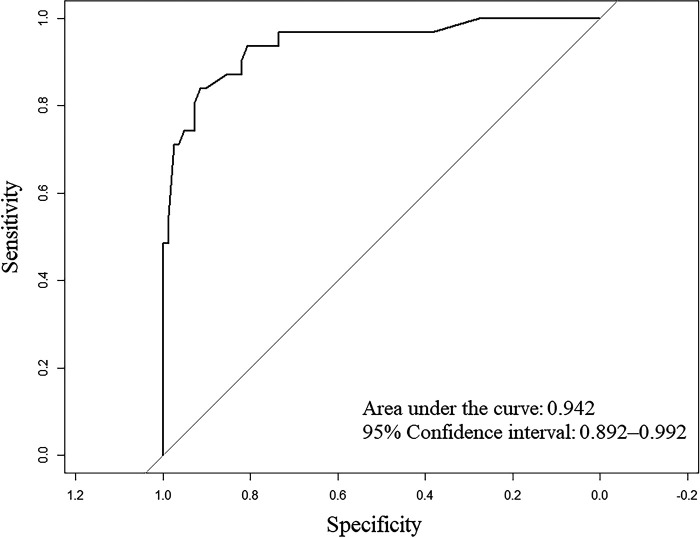
Receiver operating characteristic (ROC) curve for the multivariate predictive nomogram of the pathologic complete response rate after neoadjuvant chemotherapy in breast cancer patients.

A nomogram was developed to predict the breast cancer pCR rate after NAC, according to BMI, ALB, Cys-C, POH, HGB, PDW, MONO, and D-D (Figure 2). The predicted pCR rate was calculated by summing the scores of these eight factors. The p-value obtained from the Hosmer–Lemeshow test was 0.963, which indicated that the nomogram had a good statistical fit. The internal validation curve (Figure 3) and external validation curve (Figure 4) showed a trend that the true value and the predicted value were consistent. And the C-index of external validation was 0.831(95% confidence interval 0.815–0.858),which proving that the nomogram was well-calibrated. In the ROC curve analysis, the AUC of the nomogram was 0.942 (95% confidence interval 0.892–0.992, Figure 5). When the prediction probability of pCR is 33.9%, the model has the optimal cut-off value, with a sensitivity of 83.9%, a specificity of 91.6%, and a C-index of 0.942, all of which proved that the nomogram had good discriminative and predictive abilities.

## Discussion

It is well-recognized that the therapeutic response and prognosis of tumors are not only related to the histopathological characteristics of the tumor itself, but also the state of the host (16). In this study, we analyzed the correlation between several clinical characteristics, hematological indicators, and the pCR. The hematological indicators included a total of 61 clinical laboratory indicators, and covered almost all of the hematological markers commonly measured in the clinic. Notably, these indicators not only showed the clinicopathological features of tumors, but reflected the multi-system function of patients at initial diagnosis. The nomogram was established according to eight independent predictors, namely BMI, ALB, Cys-C, POH, HGB, PDW, MONO, and D-D. The AUC of the ROC curve and the C-index indicated that the nomogram had good discriminative ability, while the Hosmer–Lemeshow test and the calibration curve proved that the nomogram was well-calibrated. These results confirm that our nomogram exhibits a high predictive value. As far as we know, this was the first attempt to predict NAC after the pCR in patients with breast cancer through comprehensively analyzing the clinical characteristics and hematological indicators that reflect the function of multiple human systems.

For breast cancer patients in China, one study found that ER,PR, HER2 and primary tumor response were associated with axillary pCR. The C index of the model training group and validation group were 0.834 and 0.756 respectively (10). Guo et al. confirmed that menopausal status, family history, initial tumor size, ER, HER2 status and Ki-67 were associated with pCR. The AUC value of this model is 0.762 (9). The reasons for different predictors may be related to population selection and different pCR definitions, namely breast pCR and axillary pCR.Among laboratory indicators related to pCR in China’s breast cancer patients, Chen et al showed that the ratio of neutrophils to lymphocytes is related to axillary pCR (21). The ratio of lymphocyte to monocyte, fibrinogen level and D-D level have been reported to predict the pCR possibility in three negative breast cancer (22). The mechanism between laboratory indexes and NAC efficacy in different subtypes needs further research.

The nutritional status of patients is closely related to the therapeutic responsiveness and prognosis of breast cancer (23). In our study, we found that patients with a lower BMI, a higher ALB concentration, and a higher HGB concentration had a better response to NAC, which proves that obesity, hypoalbuminemia, and anemia are all risk factors for the pCR. The mechanism can be explained by the imbalance in nutritional status, which can stimulate local and circulating proinflammatory cytokines and tumor stem cells, promoting tumor angiogenesis and driving tumor growth, invasion, and metastasis (24).

The formation of nucleotides, membrane phospholipids, and phosphorylation intermediates in cell signaling require the participation of inorganic P. Rapidly proliferating cells, such as tumor cells, also need large amounts of P-rich ribosomes and RNA to synthesize proteins (25, 26). Therefore, it is theoretically believed that an increase in serum P indicates that tumor cells are rapidly dividing and proliferating, which is why patients with a high P concentration have a poor response to NAC treatment.

Cysteine proteinase is an important matrix metalloproteinase that acts on the cell membrane and assists the active invasion and metastasis of tumor cells (27). As one of the potent effective cysteine proteinase inhibitors in the human body, Cys-C limits the malignant behavior of tumors (28), and an increase in Cys-C has been reported in a number of patients with cancer (29–31). Similar conclusions have been reached in our study, which suggests that patients with a higher Cys-C value have a higher possibility of achieving a pCR.

In our study, patients with a high level of PDW were less likely to achieve a pCR. A high PDW value indicates an increase in the number of immature platelets in the circulatory system, which may be related to the upregulation of interleukin-6, tumor necrosis factor-α, interleukin-1, and other pro-inflammatory cytokines that promote the maturation of heterologous megakaryocytes during tumor development (32).

The activation of innate immunity is a possible mechanism of the correlation between the number of monocytes and the response to chemotherapy and tumor prognosis (33). Monocyte lines increase through upregulation of monocyte chemoattractant protein 1 in tumors (34), and differentiate into tumor-related macrophages, which promote invasion and metastasis of tumor cells by regulating the poor response to NAC.

D-D is a degradation product of fibrin induced by plasmin (35). D-D is considered to be a biomarker of hemostasis and fibrinolysis activation and is related to the prognosis of breast cancer (36). Interestingly, contrary to a previous study, we found that patients with a higher D-D concentration were more likely to achieve a pCR. The possible mechanism underpinning this phenomenon is that patients with breast cancer have a hypercoagulable blood state and therefore may be more responsive to NAC.

Inevitably, our study has some limitations that should be highlighted. First, as a retrospective analysis performed at a single center, the number of patients involved was relatively small; thus, it is necessary to conduct external validation in large-scale multicenter research in the future to evaluate our model. Second, factors considered to be related to the pCR in some research, such as tumor stage, molecular typing (37), and Ki-67 index (38), were not confirmed in our study or proven to be independent predictors of the pCR. This may be related to the fact that indicators are not mutually independent or the population selection bias due to single-center study. The specific mechanism needs to be further explored. Third, some of the clinical characteristics of the study population were unbalanced. For example, we had a large proportion of subjects with HER2-positive molecular typing, but no patients with luminal A breast cancer. This bias is possibly because NAC is only suitable for the treatment of certain types of breast cancer. Fourthly, despite our strict exclusion and inclusion criteria, we still cannot avoid the impact of patients’ potential diseases on the results.

## Conclusion

In conclusion,in this study, we identified eight predictors independently associated with the pCR. We constructed a nomogram model with a prediction accuracy of 0.942. This nomogram can help clinicians easily predict the possibility of patients achieving a pCR after NAC and may aid in the development of effective treatment strategies.

## Data Availability

The raw data supporting the conclusions of this article will be made available by the authors, without undue reservation.
