# Supplementary material for: Development and Validation of a Nomogram to Predict the Probability of Breast Cancer Pathologic Complete Response after Neoadjuvant Chemotherapy: A Retrospective Cohort Study
Source: Front Surg. 2022 Jun 9;9:878255. doi: 10.3389/fsurg.2022.878255 (PMC9218360; doi:10.3389/fsurg.2022.878255)
Supplement: Supplementary file 1 [file Table_1_v1.docx]

Supplementary Table 1: Clinical characteristics of validation group with breast cancer and their correlation with the pathologic complete response rate after neoadjuvant chemotherapy.

| **Factors** | **Total(%)** | **pCR(%)** | **Non-pCR(%)** | **P value** |
| --- | --- | --- | --- | --- |
| Age (years, mean ± SD) | 49.2±8.6 | 48.1±9.1 | 50.3±4.2 | 0.912 |
| Clinical stage |  |  |  | 0.051 |
| Ⅱ | 80(78.4) | 21(26.3) | 59(73.8) |  |
| Ⅲ | 22(21.6) | 3(13.6) | 19(86.4) |  |
| Clinical tumor stage |  |  |  | 0.136 |
| cT1 | 12(11.8) | 2(16.7) | 10(83.3) |  |
| cT2 | 76(74.5) | 18(23.7) | 58(76.3) |  |
| cT3 | 11(10.8) | 4(36.4) | 7(63.6) |  |
| cT4 | 3(2.9) | 1(33.3) | 2(66.7) |  |
| Clinical nodal stage |  |  |  | 0.158 |
| cN0 | 9(8.8) | 4(44.4) | 5(55.6) |  |
| cN1 | 70(68.6) | 21(30.0) | 49(70.0) |  |
| cN2 | 18(17.6) | 4(22.2) | 14(77.8) |  |
| cN3 | 5(4.9) | 2(40.0) | 3(60.0) |  |
| Chemotherapy regimen |  |  |  | 0.379 |
| TAC/ AC-T/ TA | 43(42.2) | 21(48.8) | 22(51.2) |  |
| TC/TX/TP/AC | 8(7.8) | 5(62.5) | 3(37.5) |  |
| AC-TH/ TCbH | 31(30.4) | 15(48.4) | 16(51.6) |  |
| TCbHP/ THP/ AC-THP | 20(19.6) | 10(50.0) | 10(50.0) |  |
| Chemotherapy cycle |  |  |  |  |
| <6 | 11(10.8) | 2(18.2) | 9(81.8) | 0.895 |
| ≥6 | 91(89.2) | 20(22.0) | 71(78.0) |  |
| Menopausal status |  |  |  | 0.157 |
| Premenopausal | 54(52.9) | 15(27.8) | 39(72.2) |  |
| Peri/postmenopausal | 48(47.1) | 7(14.6) | 41(85.4) |  |
| Molecular subtype |  |  |  | 0.518 |
| Luminal B (Her2−) | 5(4.9) | 2(40.0) | 3(60.0) |  |
| Luminal B (Her2+) | 32(31.4) | 9(28.1) | 23(71.9) |  |
| Her2 positive | 46(45.1) | 10(21.7) | 36(78.3) |  |
| Triple negative | 19(18.6) | 5(26.3) | 14(73.7) |  |
| Estrogen receptor |  |  |  | 0.428 |
| Negative | 46(45.1) | 15(32.6) | 31(67.4) |  |
| Positive | 56(54.9) | 10(17.9) | 46(82.1) |  |
| Progesterone receptor |  |  |  | 0.384 |
| Negative | 80(78.4) | 20(25.0) | 60(75.0) |  |
| Positive | 22(21.6) | 5(22.7) | 17(77.3) |  |
| HER-2 |  |  |  | 0.354 |
| Negative | 20(19.6) | 8(40.0) | 12(60.0) |  |
| Positive | 82(80.4) | 21(25.6) | 61(74.4) |  |
| Ki-67,% |  |  |  | 0.947 |
| <27.5 | 12(11.8) | 2(16.7) | 10(83.3) |  |
| ≥27.5 | 90(88.2) | 27(30.0) | 63(70.0) |  |
| Body mass index, kg/m^2^ |  |  |  | 0.015 |
| <21.281 | 19(18.6) | 7(36.8) | 12(63.2) |  |
| ≥21.281 | 83(81.4) | 19(22.9) | 64(77.1) |  |

NAC: Neoadjuvant chemotherapy

pCR: Pathologic complete response

HER2: Human epidermal growth factor receptor 2

Supplementary Table 2: Hematological indicators of 114 breast cancer patients and their correlation with pCR rate after NAC

| **Factors** | **Total(%)** | **pCR(%)** | **Non-pCR(%)** | **P value** |
| --- | --- | --- | --- | --- |
| **Liver function test** |  |  |  |  |
| Aspartate transaminase,U/L |  |  |  | 0.108 |
| <16.5 | 35(30.7) | 6(17.1) | 29(82.9) |  |
| ≥16.5 | 79(69.3) | 25(31.6) | 54(68.4) |  |
| Alanine transaminase,U/L |  |  |  | 0.420 |
| <13.5 | 34(29.8) | 11(32.4) | 23(67.6) |  |
| ≥13.5 | 80(70.2) | 20(25.0) | 60(75.0) |  |
| Aspartate transaminase/Alanine transaminase | |  |  | 0.155 |
| <0.85 | 25(21.9) | 4(16.0) | 21(84.0) |  |
| ≥0.85 | 89(78.1) | 27(30.3) | 62(69.7) |  |
| Alkaline phosphatase,U/L |  |  |  | **0.083** |
| <61.5 | 32(28.1) | 5(15.6) | 27(84.4) |  |
| ≥61.5 | 82(79.9) | 26(31.7) | 56(68.3) |  |
| Gamma glutamyl transpeptidase,U/L | |  |  | 0.255 |
| <15.5 | 65(57.0) | 15(23.1) | 50(76.9) |  |
| ≥15.5 | 49(43.0) | 16(32.7) | 33(67.3) |  |
| Total cholesterol,mmol/L |  |  |  | 0.167 |
| <4.875 | 73(64.0) | 23(31.5) | 50(68.5) |  |
| ≥4.875 | 41(36.0) | 8(19.5) | 33(80.5) |  |
| Total bilirubin,μmol/L |  |  |  | 0.228 |
| <7.95 | 16(14.0) | 2(12.5) | 14(87.5) |  |
| ≥7.95 | 98(86.0) | 29(29.6) | 69(70.4) |  |
| Direct bilirubin,umol/L |  |  |  | 0.112 |
| <3.45 | 58(50.9) | 12(20.7) | 46(79.3) |  |
| ≥3.45 | 56(49.1) | 19(33.9) | 37(66.1) |  |
| Indirect bilirubin,μmol/L |  |  |  | 0.335 |
| <7.65 | 45(39.5) | 10(22.2) | 35(77.8) |  |
| ≥7.65 | 69(60.5) | 21(30.4) | 48(69.6) |  |
| Total protein,g/L |  |  |  | **0.073** |
| <79.45 | 90(78.9) | 21(23.3) | 69(76.7) |  |
| ≥79.45 | 24(21.1) | 10(41.7) | 14(58.3) |  |
| Albumin,g/L |  |  |  | **0.031** |
| <47.45 | 83(72.8) | 18(21.7) | 65(78.3) |  |
| ≥47.45 | 31(27.2) | 13(41.9) | 18(58.1) |  |
| Globulin,g/L |  |  |  | 0.282 |
| <33.15 | 92(80.7) | 23(25.0) | 69(75.0) |  |
| ≥33.15 | 22(19.3) | 8(36.4) | 14(63.6) |  |
| Albumin/globulin |  |  |  | 0.523 |
| <1.85 | 100(87.7) | 26(26.0) | 74(74.0) |  |
| ≥1.85 | 14(12.3) | 5(35.7) | 9(64.3) |  |
| **Kidney function test** |  |  |  |  |
| Blood Urea nitrogen,mmol/L |  |  |  | 0.118 |
| <5.01 | 72(63.2) | 16(22.2) | 56(77.8) |  |
| ≥5.01 | 42(36.8) | 15(35.7) | 27(64.3) |  |
| Creatinine,μmol/L |  |  |  | **0.069** |
| <43.5 | 24(21.2) | 3(12.5) | 21(87.5) |  |
| ≥43.5 | 90(78.9) | 28(31.1) | 62(68.9) |  |
| Cystatin C,mg/L |  |  |  | **<0.001** |
| <1.05 | 104(91.2) | 23(22.1) | 81(77.9) |  |
| ≥1.05 | 10(8.8) | 8(80.0) | 2(20.0) |  |
| Glucose,mmol/L |  |  |  | 0.141 |
| <5.015 | 57(50.0) | 12(21.1) | 45(78.9) |  |
| ≥5.015 | 57(50.0) | 19(33.3) | 38(66.7) |  |
| Uric Acid,μmol/L |  |  |  | 0.204 |
| <243.5 | 40(35.1) | 8(20.0) | 32(80.0) |  |
| ≥243.5 | 74(64.9) | 23(31.1) | 51(68.9) |  |
| **Electrolyte test** |  |  |  |  |
| Potassium,mmol/L |  |  |  | **0.704** |
| <4.34 | 95(83.3) | 29(30.5) | 66(69.5) |  |
| ≥4.34 | 19(16.7) | 2(10.5) | 17(89.5) |  |
| Sodium,mmol/L |  |  |  | 0.302 |
| <140.5 | 30(26.3) | 6(20.0) | 24(80.0) |  |
| ≥140.5 | 84(73.7) | 25(29.8) | 59(70.2) |  |
| Chlorinum,mmol/L |  |  |  | 0.291 |
| <101.35 | 42(36.8) | 9(21.4) | 33(78.6) |  |
| ≥101.35 | 72(63.2) | 22(30.6) | 50(69.4) |  |
| Phosphorus,mmol/L |  |  |  | **0.010** |
| <1.135 | 66(57.9) | 24(36.4) | 42(63.6) |  |
| ≥1.135 | 48(42.1) | 7(14.6) | 41(85.4) |  |
| Calcium,mmol/L |  |  |  | **0.030** |
| <2.325 | 63(55.3) | 12(19.0) | 51(81.0) |  |
| ≥2.325 | 51(44.7) | 19(37.3) | 32(62.7) |  |
| Magnesium,mmol/L |  |  |  | 0.234 |
| <1.025 | 76(66.7) | 18(23.7) | 58(76.3) |  |
| ≥1.025 | 38(33.3) | 13(34.2) | 25(65.8) |  |
| Carbon dioxide binding rate,mmol/L | |  |  | 0.143 |
| <22.65 | 50(43.9) | 10(20.0) | 40(80.0) |  |
| ≥22.65 | 64(56.1) | 21(32.8) | 43(67.2) |  |
| Anion gap,mmol/L |  |  |  | 0.282 |
| <20 | 22(19.3) | 8(36.4) | 14(63.6) |  |
| ≥20 | 92(80.7) | 23(25.0) | 69(75.0) |  |
| **Complete blood count** |  |  |  |  |
| Red blood cell count/L |  |  |  | **0.046** |
| <5.04 | 108(94.7) | 27(25.0) | 81(75.0) |  |
| ≥5.04 | 6(5.3) | 4(66.7) | 2(33.3) |  |
| Hemoglobin,g/L |  |  |  | **0.067** |
| <147.5 | 103(90.4) | 25(24.3) | 78(75.7) |  |
| ≥147.5 | 11(9.6) | 6(54.5) | 5(45.5) |  |
| Hematocrit,% |  |  |  | 0.132 |
| <41.4 | 75(65.8) | 17(22.7) | 58(77.3) |  |
| ≥41.4 | 39(34.2) | 14(35.9) | 25(64.1) |  |
| Mean corpuscular volume, fL |  |  |  | 0.225 |
| <89.95 | 51(44.7) | 11(21.6) | 40(78.4) |  |
| ≥89.95 | 63(55.3) | 20(31.7) | 43(68.3) |  |
| Mean corpuscular hemoglobin,pg |  |  |  | 0.234 |
| <30.55 | 69(60.5) | 16(23.2) | 53(76.8) |  |
| ≥30.55 | 45(39.5) | 15(33.3) | 30(66.7) |  |
| Mean corpuscular hemoglobin concentration,g/L | | |  | **0.065** |
| <323.5 | 99(86.8) | 30(30.3) | 69(69.7) |  |
| ≥323.5 | 15(13.2) | 1(6.7) | 14(93.3) |  |
| Red blood cell distribution width-Coefficient of variation,% | | |  | 0.205 |
| <12.45 | 34(29.8) | 12(35.3) | 22(64.7) |  |
| ≥12.45 | 80(70.2) | 19(23.8) | 61(76.3) |  |
| Red blood cell distribution width-Standard deviation,fL | | |  | 0.300 |
| <38.85 | 95(83.3) | 24(25.3) | 71(74.7) |  |
| ≥38.85 | 19(16.7) | 7(36.8) | 12(63.2) |  |
| Platelet count,10^9/L |  |  |  | 0.204 |
| <194 | 40(35.1) | 8(20.0) | 32(80.0) |  |
| ≥194 | 74(64.9) | 23(31.1) | 51(68.9) |  |
| Platelet distribution width,fL |  |  |  | **0.028** |
| <14.85 | 58(50.9) | 21(36.2) | 37(63.8) |  |
| ≥14.85 | 56(49.1) | 10(17.9) | 46(82.1) |  |
| Mean platelet volume,fL |  |  |  | **0.051** |
| <10.7 | 34(29.8) | 5(14.7) | 29(85.3) |  |
| ≥10.7 | 80(70.2) | 26(32.5) | 54(67.5) |  |
| Platelet large cell ratio,% |  |  |  | **0.068** |
| <31..5 | 37(32.5) | 6(16.2) | 31(83.8) |  |
| ≥31.5 | 77(67.5) | 25(32.5) | 52(67.5) |  |
| Thrombocytocrit,% |  |  |  | 0.258 |
| <0.245 | 54(47.4) | 12(22.2) | 42(77.8) |  |
| ≥0.245 | 60(52.6) | 19(31.7) | 41(68.3) |  |
| White blood cell count,10^9/L |  |  |  | 0.360 |
| <4.675 | 25(21.9) | 5(20.0) | 80(80.0) |  |
| ≥4.675 | 89(78.1) | 26(29.2) | 63(70.8) |  |
| Lymphocyte count,10^9/L |  |  |  | 0.227 |
| <1.445 | 52(45.6) | 17(32.7) | 35(67.3) |  |
| ≥1.445 | 62(54.4) | 14(22.6) | 48(77.4) |  |
| Monocyte count,10^9/L |  |  |  | **0.004** |
| <0.185 | 15(13.2) | 9(60.0) | 6(40.0) |  |
| ≥0.185 | 99(86.8) | 22(22.2) | 77(77.8) |  |
| Neutrophil count,10^9/L |  |  |  | 0.189 |
| <4.08 | 70(61.4) | 16(22.9) | 54(77.1) |  |
| ≥4.08 | 44(38.6) | 15(34.1) | 29(65.9) |  |
| Eosinophil count,10^9/L |  |  |  | 0.224 |
| <0.025 | 31(27.2) | 11(35.5) | 20(64.5) |  |
| ≥0.025 | 83(72.8) | 20(24.1) | 63(75.9) |  |
| Basophil count,10^9/L |  |  |  | 0.135 |
| <0.025 | 72(63.2) | 23(31.9) | 49(68.1) |  |
| ≥0.025 | 42(36.8) | 8(19.0) | 34(81.0) |  |
| Lymphocyte percentage,% |  |  |  | 0.448 |
| <24.75 | 45(39.5) | 14(31.1) | 31(68.9) |  |
| ≥24.75 | 69(60.5) | 17(24.6) | 52(75.4) |  |
| Monocyte percentage,% |  |  |  | **0.020** |
| <3.55 | 21(18.4) | 10(47.6) | 11(52.4) |  |
| ≥3.55 | 93(81.6) | 21(22.6) | 72(77.4) |  |
| Neutrophil percentage,% |  |  |  | 0.160 |
| <72.65 | 81(71.1) | 19(23.5) | 62(76.5) |  |
| ≥72.65 | 33(28.9) | 12(36.4) | 21(63.6) |  |
| Eosinophil percentage,% |  |  |  | 0.275 |
| <0.85 | 53(46.5) | 17(32.1) | 36(67.9) |  |
| ≥0.85 | 61(53.5) | 14(23.0) | 47(77.0) |  |
| Basophil percentage,% |  |  |  | 0.103 |
| <0.35 | 52(45.6) | 18(34.6) | 34(65.4) |  |
| ≥0.35 | 62(54.4) | 13(21.0) | 49(79.0) |  |
| **Coagulation function test** |  |  |  |  |
| Prothrombin time,S |  |  |  | 0.570 |
| <13.15 | 71(62.3) | 18(25.4) | 53(74.6) |  |
| ≥13.15 | 43(37.7) | 13(30.2) | 30(69.8) |  |
| Prothrombin activity |  |  |  | 0.354 |
| <105.95 | 77(67.5) | 23(29.9) | 54(70.1) |  |
| ≥105.95 | 37(32.5) | 8(21.6) | 29(78.4) |  |
| Prothrombin ratio |  |  |  | 0.308 |
| <1.015 | 64(56.1) | 15(23.4) | 49(76.6) |  |
| ≥1.015 | 50(43.9) | 16(32.0) | 34(68.0) |  |
| Prothrombin International normalized ratio | |  |  | 0.156 |
| <1.075 | 103(90.4) | 30(29.1) | 73(70.9) |  |
| ≥1.075 | 11(9.6) | 1(9.1) | 10(90.9) |  |
| Activated partial thromboplastin time,S | |  |  | **0.088** |
| <35.95 | 59(51.8) | 12(20.3) | 47(79.7) |  |
| ≥35.95 | 55(48.2) | 19(34.5) | 36(65.6) |  |
| Activated partial thromboplastin ratio | |  |  | 0.141 |
| <1.055 | 57(50.0) | 12(21.1) | 45(78.9) |  |
| ≥1.055 | 57(50.0) | 19(33.3) | 38(66.7) |  |
| Thrombin time,S |  |  |  | **0.013** |
| <16.75 | 75(65.8) | 26(34.7) | 49(65.3) |  |
| ≥16.75 | 39(34.2) | 5(12.8) | 34(87.2) |  |
| Thrombin time ratio |  |  |  | **0.013** |
| <0.985 | 75(65.8) | 26(34.7) | 49(65.3) |  |
| ≥0.985 | 39(34.2) | 5(12.8) | 34(87.2) |  |
| Fibrinogen content,g/L |  |  |  | **0.022** |
| <3.085 | 64(56.1) | 12(18.8) | 52(81.3) |  |
| ≥3.085 | 50(43.9) | 19(38.0) | 31(62.0) |  |
| D-dimer,mg/L |  |  |  | **<0.001** |
| <0.55 | 83(72.8) | 15(18.1) | 68(81.9) |  |
| ≥0.55 | 31(27.2) | 16(51.6) | 15(48.4) |  |
| Fibrinogen degradation products,mg/L | |  |  | 0.147 |
| <0.755 | 18(15.8) | 2(11.1) | 16(88.9) |  |
| ≥0.755 | 96(84.2) | 29(30.2) | 67(69.8) |  |
| NAC: Neoadjuvant chemotherapy |  |  |  |  |
| pCR: Pathological complete response | |  |  |  |
